# New Chromane-Based Derivatives as Inhibitors of *Mycobacterium tuberculosis* Salicylate Synthase (MbtI): Preliminary Biological Evaluation and Molecular Modeling Studies

**DOI:** 10.3390/molecules23071506

**Published:** 2018-06-21

**Authors:** Elena Pini, Giulio Poli, Tiziano Tuccinardi, Laurent Roberto Chiarelli, Matteo Mori, Arianna Gelain, Luca Costantino, Stefania Villa, Fiorella Meneghetti, Daniela Barlocco

**Affiliations:** 1 Dipartimento di Scienze Farmaceutiche, Università degli Studi di Milano, Via L. Mangiagalli 25, 20133 Milano, Italy; elena.pini@unimi.it (E.P.); matteo.mori@unimi.it (M.M.); arianna.gelain@unimi.it (A.G.); stefania.villa@unimi.it (S.V.); daniela.barlocco@unimi.it (D.B.); 2 Dipartimento di Farmacia, Università di Pisa, via Bonanno 6, 56126 Pisa, Italy; giulio.poli@unipi.it (G.P.); tiziano.tuccinardi@unipi.it (T.T.); 3 Dipartimento di Biologia e Biotecnologie “Lazzaro Spallanzani”, Università degli Studi di Pavia, via Ferrata 9, 27100 Pavia, Italy; laurent.chiarelli@unipv.it; 4 Dipartimento Scienze della Vita, Università degli Studi di Modena e Reggio Emilia, via Campi 103, 41121 Modena, Italy; luca.costantino@unimore.it

**Keywords:** antimycobacterial agent, siderophore, mycobactin, iron, consensus docking, chorismate, MD simulation

## Abstract

Tuberculosis is the leading cause of death from a single infectious agent worldwide; therefore, the need for new antitubercular drugs is desperate. The recently validated target salicylate synthase MbtI is the first enzyme involved in the biosynthesis of mycobactins, compounds able to chelate iron, an essential cofactor for the survival of *Mycobacterium tuberculosis* in the host. Here, we report on the synthesis and biological evaluation of chromane-based compounds as new potential inhibitors of MbtI. Our approach successfully allowed the identification of a novel lead compound (**1**), endowed with a promising activity against this enzyme (IC_50_ = 55 μM). Molecular modeling studies were performed in order to evaluate the binding mode of **1** and rationalize the preliminary structure-activity relationships, thus providing crucial information to carry out further optimization studies.

## 1. Introduction

Tuberculosis (TB), one of the deadliest infectious diseases in the world with about 1.7 million deaths reported in 2016, is caused by *Mycobacterium tuberculosis* (Mtb) [1,2]. Antitubercular therapy has existed since the late 1940s [3], but the long duration of the prescribed treatment (6–12 months) often causes low patient compliance. Another problem is that Mtb can survive for extended periods of time in its non-replicative or persistent state, becoming resistant to conventional forms of chemotherapy. Patient non-compliance, coupled with the ability of Mtb to enter the persistent state, has contributed to the alarming emergence of multidrug-resistant (MDR-TB) and extensively drug-resistant (XDR-TB) mycobacterial strains [4,5]. Mtb has developed resistance to the first-line drugs isoniazid and rifampicin (MDR-TB), and resistance against second-line injectable drugs like fluoroquinolones is a growing concern (XDR-TB) [6]. Drug-resistant TB poses a serious threat to existing control programs since the therapy becomes less effective, more complex and more expensive, compared to that of drug-susceptible forms. There are a number of novel anti-TB compounds in clinical trials, and two new drugs have been recently approved: delamanid and bedaquiline [7]. In particular, more information on the effectiveness, safety, and tolerability of bedaquiline is urgently required, because its severe side effects (mainly QT prolongation) could be potentially life-threatening [8]. Considering these issues, the identification of novel targets [9,10,11] and the development of new scaffolds to sustain the TB drug pipeline are imperative [12].

**Scheme 1:**
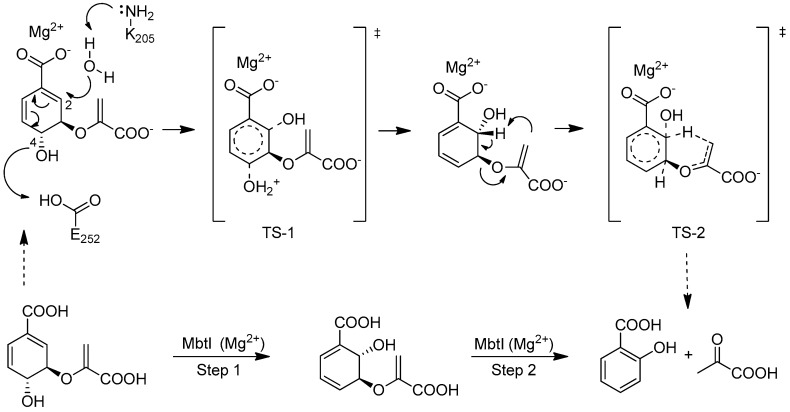
Reactions catalyzed by MbtI, the salicylate synthase of Mtb.

Mtb survival in the host is highly dependent on the availability of iron; this cofactor is made available through two different strategies: the synthesis of siderophores (mycobactins), compounds able to chelate iron, and the uptake/metabolism of host heme. Water-soluble carboxymycobactins and lipophilic mycobactins, compounds that vary according to the nature of their lipophilic moiety, are able to solubilize iron and compete with the iron-binding proteins (e.g., transferrin, lactoferrin, ferritin) of the host [9]. MbtI is the first enzyme involved in the mycobactin biosynthetic pathway; it catalyzes the interconversion of chorismate to salicylic acid, via the intermediate isochorismate [9] (Scheme 1).

This enzyme is a valuable target to develop new drugs able to counteract Mtb infection, since it has been structurally and biochemically characterized [13], and it is essential for the survival of the mycobacterium under iron-deficient conditions [14,15,16]. Moreover, it is absent in the host, making MbtI inhibitors potentially safe for humans. Several strategies have been followed for the discovery of MbtI inhibitors [17]. In particular, substrate (chorismate and isochorismate) analogues, transition state analogues and compounds originated from HTS have been identified [9,17,18].

Inspired by the work of Aldrich and co-workers [19], which focused on the synthesis of analogues of the first transition state (TS-1) of the isochorismate partial reaction (see Scheme 1), we observed that the bicyclic chromane and 4-oxochromane motifs, if bearing a carboxylic moiety at C5 position, could be suitable scaffolds to mimic the transition states formed in the reaction catalyzed by MbtI. In particular, the chromanone nucleus caught our attention as a privileged bicyclic scaffold in medicinal chemistry and core structural element of compounds endowed with antitubercular activity [20,21]. Moreover, the chroman-4-one framework has been reported to be an integral part of several antimycobacterial agents [21,22,23]. Encouraged by these data, we addressed our efforts to the research of new MbtI inhibitors bearing this scaffold. In this work, we assayed against MbtI compounds **1**–**5**, chromane and chromanone derivatives available in our in-house library. The best inhibitor **1** demonstrated a promising activity, which was investigated by means of computational tools. This encouraging result could pave the way for further improvements on this promising scaffold.

## 2. Results and Discussion

### 2.1. Synthesis

**Scheme 2:**
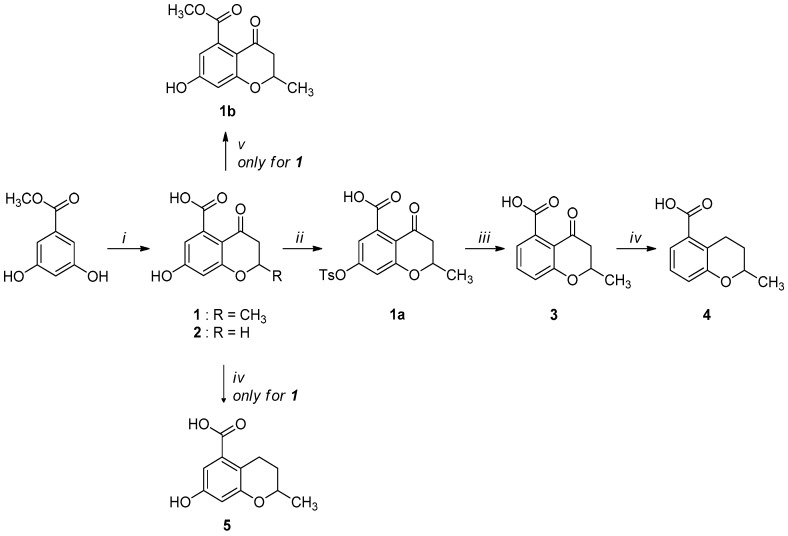
Synthetic route for compounds **1**–**5**. Reagents and conditions: (*i*) a. CH_3_CH=CHCOCl or CH_2_=CHCOCl, BF_3_Et_2_O, PhNO_2_, N_2_, 24 h, reflux; b. HCl, overnight, 0 °C; c. water, steam-distillation; yield: 40% for **1**; yield: 35% for **2**; (*ii*) NaOH, TsOCl, THF, overnight, rt; yield: 82%; (*iii*) Ni Raney, NaHCO_3_, water; overnight, rt; quantitative yield; (*iv*) Pd/BaSO_4_, H_2_, ethanol, 6 h, 5 atm., rt; yield: 70% for **4**; 75% for **5**; (*v*) CH_3_OH, conc. H_2_SO_4_, 24 h, reflux; quantitative yield.

Compounds **1**–**5** were obtained through the synthetic route outlined in detail in Scheme 2. Their synthesis started from the condensation of methyl 3,5-dihydroxybenzoate and crotonoyl chloride or acryloyl chloride in the presence of boron trifluoride etherate as base, which led to chromanones **1** and **2**, respectively. Successively, the hydroxyl group was quantitatively converted into the corresponding *p*-toluenesulfonyl derivative **1a**, using sodium hydroxide and *p*-toluenesulfonyl chloride [24]. The elimination of the *O*-tosyl group was performed using Ni Raney, according to the method described by Kenner and Murray [25], affording the intermediate **3**. The isolation of carboxylchromane **4**, though in a low yield, was performed by hydrogenation in the presence of Pd/BaSO_4_ [26], since the more frequently used reduction with Pd/C [27] and the Clemmensen reaction [28] were too drastic. Unsuccessfully, we also tried other methods involving the use of zinc acetate, zinc trifluoroacetate, tin/HCl, the Wolff-Kishner reduction with hydrazine and a zinc-aluminum chloride hexahydrate-THF-water system, a promising chemoselective green reagent and catalyst in organic synthesis [29]. The hydroxyl chromane derivative **5** was obtained in the same reductive condition used for **4**, starting from **1**. Finally, the methyl ester (**1b**) of **1** was obtained according to a common procedure (Fischer-Speier esterification).

### 2.2. Biological Evaluation and Preliminary Structure-Activity Relationships (SAR)

The chromane and chromanone derivatives reported in Table 1 were submitted to biological evaluation to assess their MbtI inhibitory activity. The results were compared with that of one of the most potent MbtI inhibitor reported to date, (1-carboxyprop-1-enyloxy)-2-hydroxybenzoic acid (**I**) [9], synthesized following the procedure reported in the literature [18] and used as a positive control.

**Figure 1:**
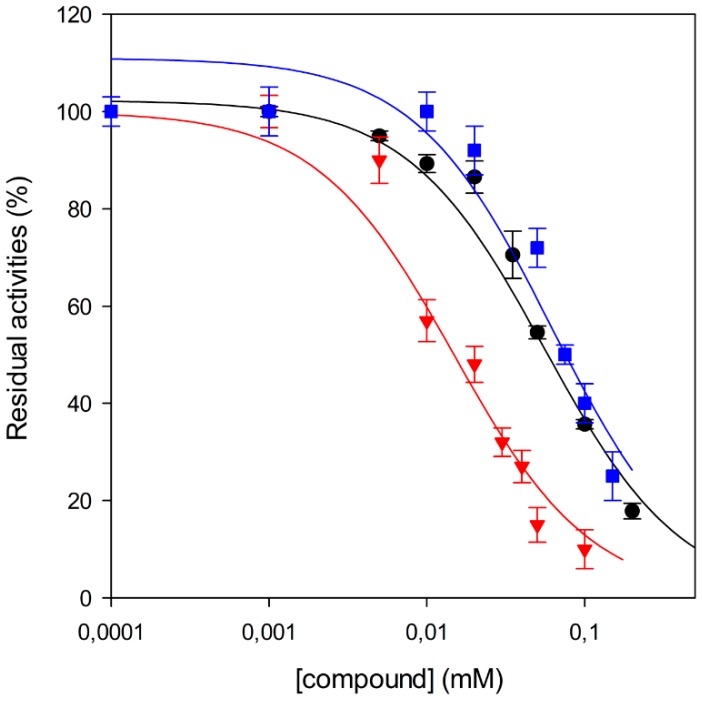
Effects of **1** (●) and **2** (■) against MbtI enzyme activity compared with **I** (▼). IC_50_ values were determined by assaying the enzyme at subsaturating concentrations of chorismic acid (50 μM).

Among the tested compounds, **1** revealed a remarkable potency, showing more than 75% inhibition of MbtI catalytic activity at a concentration of 100 μM and an IC_50_ of 55.8 ± 4.2 μM; therefore, **1** is less than 5-fold less potent than the reference **I**. The absence of the methyl group in **2** showed that this substituent did not influence the inhibitory effect, as demonstrated by the similar IC_50_ values of **1** and **2** (Figure 1).

Since the 7-hydroxyl function was demonstrated to be a key structural element to enhance the antibacterial activity of the 4-chromanone nucleus [30], we tested the chromanone **3** and chromane **4**, both lacking the 7-hydroxyl group. They displayed very weak activities, with less than 25% inhibition of MbtI catalytic activity at 100 μM, showing that this group plays a key role for MbtI inhibition too. Previously, it was found that small and polar functionalities at the 4-position of the chromane scaffold played important roles against Mtb [24]. Our finding showed that the presence of the carbonyl group in the chromanone scaffold has not a prominent role in retaining MbtI inhibitory potency. As reported in Table 1, compound **5** showed a halved inhibitory activity compared to **1**. In order to assess if the carboxylic group was essential for the activity of the best candidate **1**, the corresponding ester derivative **1b** was synthesized and tested. This compound showed a very low inhibitory activity on MbtI, thus confirming the importance of the interaction between this function and the target enzyme [9].

### 2.3. Binding Mode Evaluation of ***1***–***5*** within the MbtI Catalytic Site

**Figure 2:**
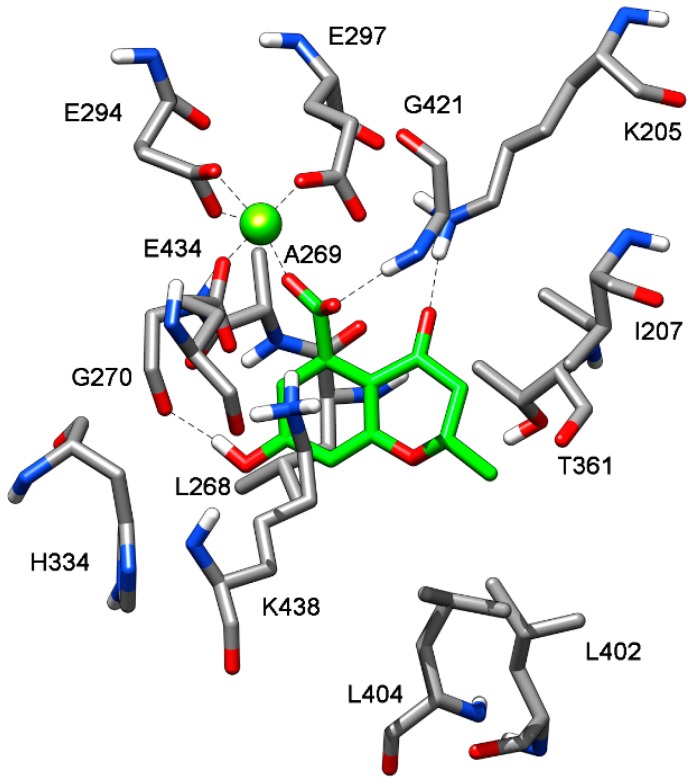
Minimized average structure of **1** docked at MbtI binding site.

To suggest a binding mode for this class of derivatives, the interaction of **1** with MbtI was analyzed by means of docking and molecular dynamics (MD) simulations. As a first step, a consensus docking method was applied as it is known to predict the ligand-binding pose better than single docking programs [31]. By using this kind of approach, a ligand is docked into the target protein by means of different docking procedures. Then, among the different best-ranked poses (originated by the different docking methods) the pose in common with the largest number of docking procedures is considered as the best docking pose. The **1**-MbtI complex obtained by means of this docking strategy was then subjected to 100 ns of MD simulation with explicit water molecules, as described in the Experimental Section. Figure 2 shows the main interactions between **1** and MbtI: the carboxylic group of the compound chelates the magnesium ion and it is involved in an H-bond with the nitrogen backbone of G241, while the aromatic ring shows a cation-π interaction with K438, that is also present in the crystallographic conformation of **I** inside the binding pocket [18]. The hydroxyl group of **1** forms a stable H-bond with the oxygen backbone of G270 that cannot be formed by **I**, which however shows a π-π interaction between the close residue H334 and its alkene moiety (see Figure S1). Regarding the interactions of the 2-methyl-2*H*-pyran-4(3*H*)-one scaffold of **1**, the carbonyl oxygen shows an H-bond with the charged nitrogen of K205, the methyl substituent shows lipophilic interactions with the side chains of L402 and L404, whereas the oxygen of the bicyclic ring does not seem to establish important interactions. The chromanone **1** is thus able to form two different H-bonds, which are not observed in the MbtI-**I** complex. Nevertheless, **I** is able to better fit into the catalytic pocket of MbtI, forming additional van der Waals interactions with the residues surrounding its aromatic ring. Moreover, thanks to the presence of an additional carboxylic group, the ligand can establish a further H-bond interaction with Y385 (Figure S1).

**Figure 3:**
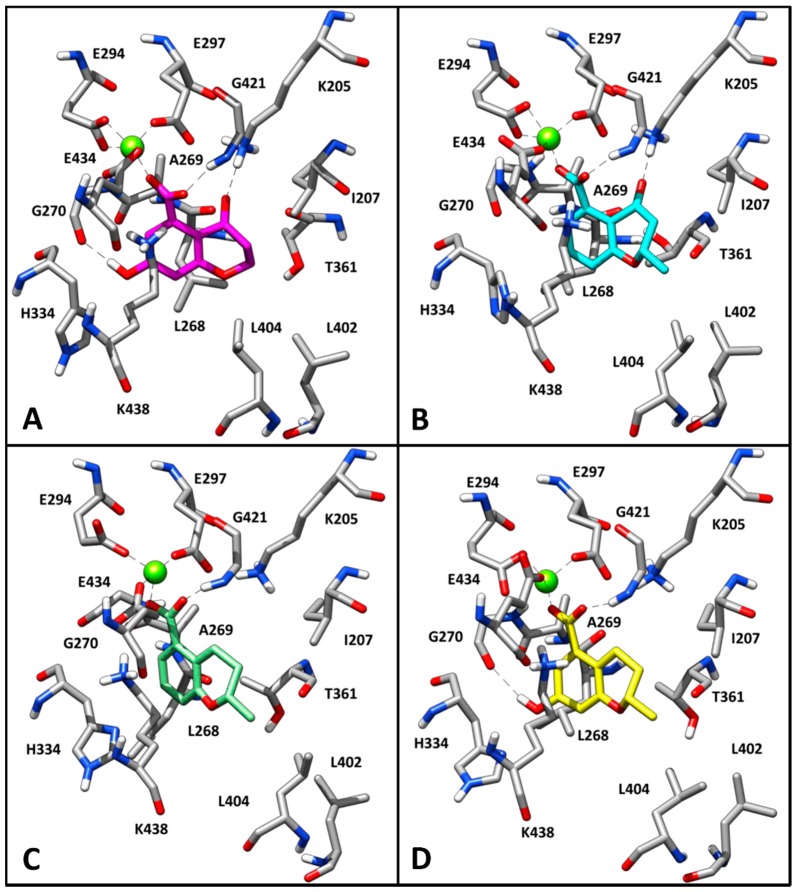
Minimized average structure of compounds **2** (**A**), **3** (**B**), **4** (**C**) and **5** (**D**) docked into MbtI binding site.

In order to better evaluate the binding mode of this series of compounds in relation to the derived SAR, the same consensus docking and MD simulation protocols applied to **1** were also used to predict the putative binding disposition of **1b**–**5** within the MtbI catalytic site. As shown in Figure 3, **2**–**5** are predicted to assume a similar binding mode, with their carboxylic moiety chelating the magnesium ion and H-bonding G421, and with their bicyclic scaffold predominantly sandwiched between K438 from one side and T361 from the other side. All compounds form a cation-π interaction with K438 and show hydrophobic contacts with the methyl group of T361, as well as with L404. This result suggests that all these derivatives are able to bind to the enzyme due to the presence of the fundamental carboxylic group. However, **3** and **4**, which cannot form the H-bond with G270 in their predicted binding mode as they lack the hydroxyl group (Figure 3B,C), displayed only a minimal activity. Interestingly, although **3** is predicted to form an H-bond with K205, it shows a comparable activity to **4**, suggesting that the carbonyl group contributes only marginally to the activity. This supports the SAR data highlighting that the 7-hydroxyl group represents another key structural element, together with the carboxylic group, to enhance the inhibitory activity of the ligands. Indeed, **5**, which does not interact with K205 but forms an H-bond with G270 through the 7-hydroxyl group, maintains considerable inhibitory activity compared to **1**. Finally, as expected on the basis of the predicted binding mode of **1**, the 2-methyl group of the chromanone scaffold does not contribute to the affinity of the enzyme, since **2**, which showed a binding mode very similar to **1**, retained total inhibitory activity. On the contrary, **1b** was not predicted to assume the binding mode shown by its analogues. This compound is not able to chelate the magnesium ion and thus it shows a completely reversed binding disposition, where the key H-bonds formed by **1** are lost (see Figure S2).

## 3. Materials and Methods

### 3.1. General Chemistry

Commercial chemicals and solvents were of reagent grade, purchased from suppliers (Sigma-Aldrich, St. Louis, MO, USA) and used as received. Anhydrous solvents were used without further drying. Silica gel plates on glass support (0.25 nm, Sigma-Aldrich) were used to follow the course of the reactions and visualized under UV lamp operating at wavelengths of 254 or 365 nm; when necessary, the spots were evidenced using a KMnO_4_ solution. Silica gel 60 (Sigma-Aldrich pore size 60 Å, 63–200 μm) was used for the purification of intermediates and final compounds. All tested compounds were characterized by the means of FT-IR, NMR, and MS to check their purity. ^1^H and ^13^C NMR and bidimensional analyses were acquired at ambient temperature with a Varian-Oxford 300 MHz instrument, operating at 300 MHz for ^1^H and 75 MHz for ^13^C. Chemical shifts are expressed in ppm (δ) from tetramethylsilane resonance in the indicated solvent (TMS: δ = 0.0 ppm), while *J*-couplings are given in Hertz. The APT sequence was used to distinguish methine and methyl carbon signals from those of methylene and quaternary carbons. FT-IR spectra were collected using the Spectrum One Perkin Elmer (Waltham, MA, USA) FT-IR Spectrometer in the spectral region between 4000 and 600 or 450 cm^−1^ for solid or liquid compounds, respectively, and analyzed by transmittance technique with 32 scans and 4 or 8 cm^−1^ resolution. Solid samples were mixed in a mortar with KBr (1:100) and pressed by a hydraulic press (10 tons) into small tablets. For liquid samples one drop was placed between two plates of sodium chloride. MS analyses were carried out with a Thermo Finnigan (Waltham, MA, USA) LCQ Advantage system, equipped with a quaternary pump, a Diode Array Detector (working wavelength: 254 nm) and a MS spectrometer, with an Electrospray ionization source and an Ion Trap mass analyzer (ionization: ESI positive or ESI negative; capillary temperature: 250 °C; source voltage: 5.50 kV; source current: 4.00 μA; multipole 1 and 2 offset: −5.50 V and −7.50 V respectively; intermultipole lens voltage: −16.00 V; trap DC offset voltage: −10.00 V). The purity of the compounds was assessed by means of high-resolution mass spectrometry using a Waters SYNAPT G2 Q-TOF mass spectrometer.

#### 3.1.1. Synthesis of 7-Hydroxy-2-methyl-4-oxo-3,4-dihydro-2*H*-benzopyran-5-carboxylic acid (**1**) and of 7-Hydroxy-4-oxo-3,4-dihydro-2*H*-benzopyran-5-carboxylic acid (**2**)

A mixture of methyl 3,5-dihydroxybenzoate (2.0 g, 11.8 mmol), crotonoyl chloride (for **1**) or acryloyl chloride (for **2**) (19.0 mmol), boron trifluoride etherate (4.5 mL, 35.7 mmol) and nitrobenzene (12 mL) was heated at 85 °C for 24 h, under nitrogen atmosphere. Then, the black mixture was poured into 5 M HCl (60 mL) and nitrobenzene was removed by steam distillation. The hot tarry aqueous distillation residue was filtered, and 5 M HCl (24 mL) was added to the clear red filtrate before cooling the mixture in an ice-bath. The solution was then extracted with diethyl ether (3 × 25 mL), and the combined organic layers were dried over anhydrous Na_2_SO_4_, filtered and evaporated in vacuo giving the crude products.

Compound **1** was obtained as a partially crystalline orange-brown solid. Crystallization from ethyl acetate/light petroleum ether gave pink crystals. Yield 40%, m.p. 251–252 °C. ^1^H-NMR (300 MHz, DMSO-*d*_6_): δ 12.81 (broad s. exch. D_2_O, 1H, COOH), 10.79 (broad s. exch. D_2_O, 1H, OH), 6.32 (s, 2H, H_6_, H_8_), 4.59–4.57 (m, 1H, H_2_), 2.58–2.56 (m, 2H, H_3_, H_3′_), 1.38 (d, 3H, *J* = 6.2 Hz, CH_3_) ppm. ^13^C-NMR (75 MHz, DMSO-*d*_6_): δ 189.75, 170.61, 164.29, 163.81, 138.41, 110.53, 109.32, 103.60, 74.85, 44.28, 21.10 ppm. FT-IR (KBr): ν 3320, 2994, 2913, 1709, 1665, 1600, 1581, 1420, 1294, 1229, 1164 cm^−1^. MS (ESI): *m*/*z* calcd for C_11_H_10_O_5_ 222.19 found *m*/*z* 221.28 [M − H]^−^.

HRMS (ESI-QTOF) calcd. for C_11_H_10_O_5_: [M − H]^−^ 221.0450; found: 221.0451.

Compound **2** was purified by column chromatography (ethyl acetate/methanol 8:2) to afford pink crystals. Yield 35%, m.p. (dec) 220–230 °C. ^1^H-NMR (300 MHz, DMSO-*d*_6_): δ 13.00 (broad s. exch. D_2_O, 1H, COOH), 9.90 (broad s. exch. D_2_O, 1H, OH), 7.07 (d, 1H, *J* = 2.6 Hz, H_6_), 6.62 (d, 1H, *J* = 2.6 Hz, H_8_), 3.17 (t, 2H, *J* = 6.6 Hz, H_2_), 2.69 (t, 2H, *J* = 6.6 Hz, H_3_) ppm. ^13^C-NMR (75 MHz, DMSO-*d*_6_): δ 168.65, 168.02, 156.83, 153.61, 131.37, 115.54, 113.47, 107.62, 28.79, 21.01 ppm. FT-IR (KBr): ν 3319, 3081, 1746, 1688, 1625, 1588, 1405, 1287, 1264, 1168, 1150, 1129 cm^−1^. MS (ESI): *m*/*z* calcd for C_10_H_8_O_5_ 208.04, found *m*/*z* 207.74 [M − H]^−^, *m*/*z* 415.34 [2M + Na]^+^.

HRMS (ESI-QTOF) calcd. for C_10_H_8_O_5_: [M − H]^−^ 207.0293; found: 207.0296.

#### 3.1.2. Synthesis of 2-Methyl-4-oxo-7-(toluene-*p*-sulfonyloxy)-3,4-dihydro-2*H*-benzopyran-5-carboxylic acid (**1a**)

Compound **1** (0.90 g, 4.1 mmol) was dissolved in THF (11 mL) and a 15% NaOH solution (6 mL) was added. To the resulting red solution cooled at 0 °C, *p*-tosyl chloride (0,77 g, 4.0 mmol) in THF (6 mL) was added dropwise, and the reaction mixture was stirred overnight at room temperature. A 10% HCl solution (10 mL) was then added until pH 2–3 and the resulting mixture was extracted with ethyl acetate (2 × 25 mL). The combined organic layers were washed with water (2 × 10 mL) and dried over anhydrous Na_2_SO_4_, filtered and concentrated in vacuo. Crystallization from ethyl acetate/light petroleum ether gave colorless needles of **1a**. Yield 83%, m.p. 161–162 °C. ^1^H-NMR (300 MHz, DMSO-*d*_6_): δ 10.55 (broad s. exch. D_2_O, 1H, COOH), 7.81 (d, 2H, *J* = 8.0 Hz, H_3Ts_, H_5Ts_), 7.49 (d, 2H, *J* = 8.0 Hz, H_2Ts_, H_6Ts_), 6.80 (d, 1H, *J* = 2.2 Hz, H_6_), 6.63 (d, 1H, *J* = 2.2 Hz, H_8_) , 4.75–4.64 (m, 1H, H_2_), 2.83–2.63 (m, 2H, H_3_, H_3′_), 2.42 (s, 3H, CH_3Ts_), 1.38 (d, 3H, *J* = 6.2 Hz, CH_3_) ppm. FT-IR (KBr): ν 3375, 3328, 3078, 2984, 2919, 1713, 1699, 1598, 1383, 1297, 1193, 1180 cm^−1^. MS (ESI): *m*/*z* calcd for C_18_H_16_SO_7_ 376.30, found *m*/*z* 377.32 [M + H]^+^.

#### 3.1.3. Synthesis of 2-Methyl-4-oxo-3,4-dihydro-2*H*-benzopyran-5-carboxylic acid (**3**)

To a stirred solution of **1a** (1.25 g, 3.3 mmol) in 0.2 M NaHCO_3_ (75 mL), a suspension of Raney Nickel (1:5 *w*/*w*) catalyst was added. The mixture was stirred over a period of 6 h at room temperature. Then, the catalyst was filtered through a celite pad; the filtrate was acidified with 4 M HCl (15 mL) until pH 2–3 and extracted with diethyl ether (3 × 25 mL). The combined organic layers were dried over anhydrous Na_2_SO_4_, filtered and evaporated in vacuo, affording the pure **3**. Quantitative yield, m.p. 167–168 °C. ^1^H-NMR (300 MHz, DMSO-*d*_6_): δ 12.85 (broad s. exch D_2_O, 1H, COOH), 7.54 (t, 1H, *J* = 8.4, Hz, H_7_), 7.07 (d, 1H, *J* = 8.4 Hz, H_6_), 6.93 (d, 1H, *J* = 7.3 Hz, H_8_), 4.70–4.63 (m, 1H, H_2_), 2.71–2.57 (m, 2H, H_3_, H_3′_), 1.41 (d, 3H, *J* = 6.2 Hz, CH_3_) ppm. ^13^C-NMR (75 MHz, DMSO-*d*_6_): δ 191.58, 170.79, 161.76, 136.29, 136.26, 120.07, 119.41, 117.62, 74.83, 44.51, 21.05 ppm. FT-IR (KBr): ν 3338, 2979, 2918, 1703, 1683, 1596, 1484, 1298 cm^−1^. MS (ESI): *m*/*z* calcd for C_11_H_10_O_4_ 206.19 found *m*/*z* 207.87 [M + H]^+^.

HRMS (ESI-QTOF) calcd. for C_11_H_10_O_4_: [M − H]^−^ 205.0501; found: 205.0502.

#### 3.1.4. Synthesis of 2-Methyl-3,4-dihydro-2*H*-benzopyran-5-carboxylic acid (**4**)

Pd/BaSO_4_ (264 mg, 0.8 mmol) was stirred in anhydrous ethanol (5 mL), then **3** (90 mg, 0.4 mmol), previously dissolved in anhydrous ethanol (5 mL), was added. The resulting mixture was reduced with hydrogen in autoclave at 5 atm for 6 h at room temperature. After completion of the reaction, the catalyst was filtered off on a celite pad, the filter cake was rinsed with ethanol, and the filtrate was evaporated under reduced pressure. The residue was dissolved in ethyl acetate (5 mL), diluted with water (2 mL), acidified with 10% HCl (2 mL) until pH = 2, and extracted with ethyl acetate (3 × 5 mL). The combined organic layers were dried over anhydrous Na_2_SO_4_, filtered and evaporated in vacuo to give a solid residue, which was then purified by chromatographic column (ethyl acetate/methanol 9:1), to obtain **4** as an oil. Yield 70%. ^1^H-NMR (300 MHz, DMSO-*d*_6_): δ 12.64 (broad s. exch D_2_O, 1H, COOH), 7.35 (dd, 1H, *J* = 7.6, 1.3 Hz, H_6_), 7.14 (t, 1H, *J* = 7.6, Hz, H_7_), 6.92 (d, 1H, *J* = 7.6 Hz, H_8_), 4.14–4.06 (m, 1H, H_2_), 3.03 (ddd, 1H, *J* = 18.0, 5.8, 3.2 Hz, H_3_), 2.95 (ddd, 1H, *J* = 18.0, 11.5, 6.2 Hz, H_3′_), 2.05–1.96 (m, 1H, H_4_), 1.61–1.47 (m, 1H, H_4’_), 1.32 (d, 3H, *J* = 6.2 Hz, CH_3_) ppm. ^13^C-NMR (75 MHz, DMSO-*d*_6_): δ 168.93, 155.60, 131.93, 126.98, 123.45, 122.44, 120.60, 71.76, 28.91, 23.84, 21.44 ppm. FT-IR (KBr): ν 3429.8, 2968.8, 2928.8, 2645.1, 1687.9, 1594.4, 1457.8, 1278.7, 1139.9, 1109.1, 801.2 cm^−1^. MS (ESI): *m*/*z* calcd for C_11_H_12_O_3_ 192.20, found *m*/*z* 191.20 [M − H]^−^.

HRMS (ESI-QTOF) calcd. for C_11_H_12_O_3_: [M − H]^−^ 191.0708; found: 191.0709.

#### 3.1.5. Synthesis of 7-Hydroxy-2-methylchroman-5-carboxylic acid (**5**)

Pd/BaSO_4_ (0.55 g, 1.62 mmol) was stirred in anhydrous ethanol (15 mL), then **1** (0.2 g, 0.90 mmol), dissolved in 10 mL of anhydrous ethanol, was charged. The resulting mixture was stirred under hydrogen flow in autoclave at 5 atm for 6 h at room temperature. After completion of the reaction, the catalyst was filtered through a celite pad, the filter cake was rinsed with ethanol, and the filtrate was evaporated under reduced pressure. The residue was dissolved in ethyl acetate (10 mL) and then washed with 2 M HCl (3 × 5 mL). The organic layer was dried over anhydrous Na_2_SO_4_, filtered and evaporated in vacuo to give **5** as a yellow solid. Yield 75%, m.p. 168–170 °C. ^1^H-NMR (300 MHz, DMSO-*d*_6_) δ 12.64 (broad s, exch. D_2_O, 1H, COOH), 9.40 (broad s, exch. D_2_O 1H, OH), 6.82 (d, *J* = 2.6 Hz, 1H, H_6_), 6.32 (d, *J* = 2.6 Hz, 1H, H_8_), 4.21–3.87 (m, 1H, H_2_), 2.96 (dd, *J* = 5.6, 3.1 Hz, 1H, H_3_), 2.90 (dd, *J* = 5.6, 3.1 Hz, 1H, H_3′_), 2.01–1.86 (m, 1H, H_4_), 1.63–1.37 (m, 1H, H_4′_), 1.27 (d, *J* = 6.2 Hz, 3H, CH_3_) ppm.^13^C-NMR (75 MHz, DMSO-*d*_6_): δ 168.87, 156.42, 156.06, 132.18, 114.15, 110.27, 107.09, 71.79, 29.21, 23.30, 21.43 ppm. FT-IR(KBr): ν 3370, 2972, 2945, 2618, 1697, 1622, 1589, 1491, 1410,1308, 1132 cm^−1^. MS (ESI): *m*/*z* calcd for C_11_H_12_O_4_ 208.07, found *m*/*z* 207.21 [M − H]^−^.

HRMS (ESI-QTOF) calcd. for C_11_H_12_O_4_: [M − H]^−^ 207.0657; found: 207.0657.

#### 3.1.6. Synthesis of 7-Hydroxy-4-oxo-3,4-dihydro-2*H*-benzopyran-5-methyl ester (**1b**)

To a cold stirred solution of **1** (90 mg, 0.405 mmol) in anhydrous MeOH, a solution of 97% H_2_SO_4_ (43 μL) was added dropwise at 0 °C. The solution was heated at reflux overnight. Afterwards methanol was evaporated in vacuo and the residue was dissolved in a NaHCO_3_ saturated solution (5 mL) and then extracted with Et_2_O (3 × 6 mL). The combined organic layers were washed with brine, dried over anhydrous sodium sulfate, and then concentrated in vacuo to obtain a light pink powder. Yield 80%, m.p. 166–168 °C. ^1^H-NMR (300 MHz, DMSO-*d*_6_): δ 10.89 (broad s. exch. D_2_O, 1H, OH), 6.37 (s, 2H, H_6_, H_8_), 4.63–4.55 (m, 1H, H_2_), 3.73 (s, 3H, COOCH_3_), 2.62–2.56 (m, 2H, H_3,_), 1.37 (d, 3H, *J* = 6.2 Hz, CH_3_) ppm. ^13^C-NMR (75 MHz, DMSO-*d*_6_): δ 189.85, 164.15, 163.65, 136.34, 110.70, 109.51, 103.95, 94.88, 74.70, 52.68, 43.94, 20.80 ppm. FT-IR (KBr): ν 3258, 2958, 2924, 1736, 1652, 1609, 1580, 1501, 1428, 1300, 1235, 1159 cm^−1^. MS (ESI): *m*/*z* calcd for C_12_H_12_O_5_ 236.07 found *m*/*z* 235.33 [M − H]^−^.

HRMS (ESI-QTOF) calcd. for C_12_H_12_O_5_ [M − H]^−^ 235.0606; found: 235.0606.

### 3.2. MbtI Inhibitory Activity

MbtI was produced in recombinant form as previously reported [17]. Enzymatic activity was determined by measuring the production of salicylic acid, by a fluorimetric assay (Ex. = 305 nm, Em. = 420 nm), slightly modified from Vasan et al. [32]. Assays were performed at 37 °C in a final volume of 400 μL, using a Perkin-Elmer LS3 fluorimeter. The reaction mixture contained: 50 mM Hepes pH 7.5, 5 mM MgCl_2_, 1–2 μM MbtI, and the reactions were started by the addition of the substrate, chorismic acid. Inhibition assays were performed at subsaturating concentrations of chorismic acid (50 μM), in the presence of the compound at 100 μM (stock dissolved 20 mM in DMSO). Blank control was performed by adding DMSO. The solubility of the compounds was evaluated in the solution in which the biochemical assays were performed (up to 100 μM concentration).

For IC_50_ determinations, the enzyme activities were measured in the presence of compound and values were estimated according to the following equation:

$$A_{\left[1\right]}=A_{\left[0\right]}x\left(1-\frac{\left[I\right]}{\left[I\right]+{\mathrm{IC}}_{50}}\right),$$

where A_[1]_ is the enzyme activity at inhibitor concentration [I] and A_[0]_ is the enzyme activity without inhibitor.

### 3.3. Docking Calculations

The crystal structure of MbtI, in complex with the inhibitor **I** (PDB code: 3VEH) was taken from the Protein Data Bank [33]. The complex was placed in a rectangular parallelepiped water box. Magnesium ion was inserted, analyzing its disposition and interactions into the structure 2FN1 (PDB code) [34]. After adding hydrogen atoms, the protein complexed with its inhibitor was minimized using Amber14 software [35] and ff14SB force field at 300 K. The complex was placed in a rectangular parallelepiped water box, an explicit solvent model for water (TIP3P) was used and the complex was solvated with a 10 Å water cap. Sodium ions were added as counter ions to neutralize the system. Two steps of minimization were then carried out; in the first stage, we kept the protein fixed with a position restraint of 500 kcal/mol·Å^2^ and we solely minimized the positions of the water molecules. In the second stage, we minimized the entire system through 5000 steps of steepest descent followed by conjugate gradient (CG) until a convergence of 0.05 kcal/Å·mol. The ligand was built by means of Maestro [36] and was then minimized in a water environment (using the generalized Born/surface area model) by means of Macromodel [37]. The minimization was performed using the CG, the MMFFs force field, and a distance-dependent dielectric constant of 1.0 until the ligands reached a convergence value of 0.05 kcal·Å^−1^·mol^−1^. Five different docking procedures were applied and for each docking calculation only the best-scored pose was taken into account [38,39,40]. The docking calculations were carried out by using GOLD 5.1 with the ASP, CSCORE CHEMPLP and GSCORE scoring functions [41] and PLANTS [42] employing the procedures previously described [43]. Each ligand was docked into the binding site of MbtI by using the different docking procedures, then the root-mean-square deviation (RMSD) of each of these docking poses against the remaining docking results was evaluated using the rms analysis software of the GOLD suite. The most populated cluster of solutions was then considered and subjected to molecular dynamic (MD) simulations.

### 3.4. MD Simulations

All simulations were performed using AMBER, version 14 [35]. MD simulations were carried out using the ff14SB force field at 300 K in a rectangular parallelepiped water box. The TIP3P explicit solvent model for water was used. Sodium ions were added as counter ions to neutralize the system. Prior to MD simulations, two steps of minimization were carried out using the same procedure described above. Particle mesh Ewald (PME) electrostatics and periodic boundary conditions were used in the simulation. The time step of the simulations was 2.0 fs with a cutoff of 10 Å for the non-bonded interactions, while SHAKE was employed to keep all bonds involving hydrogen atoms rigid. Constant-volume periodic boundary MD was carried out for 0.5 ns, during which the temperature was raised from 0 to 300 K. Then 99.5 ns of constant pressure periodic boundary MD was carried out at 300 K using the Langevin thermostat to maintain constant the temperature of our system. All the α carbons of the protein were blocked with a harmonic force constant of 10 kcal/mol·Å^2^. General Amber force field (GAFF) parameters were assigned to the ligands, while partial charges were calculated using the AM1-BCC method as implemented in the Antechamber suite of AMBER 14.

## 4. Conclusions

A small set of compounds incorporating the chromane motif was evaluated for the inhibition of MbtI enzymatic activity. The approach successfully led to the disclosure of the hit **1** provided with an encouraging activity against MbtI (IC_50_ value of 55 μM), which is less than 5-fold lower than that showed by the reference inhibitor **I**.

On the basis of the acquired data, evidencing the importance of the carbonyl group and the 7-hydroxyl function as key structural elements to gain MbtI inhibition, we identified a novel structural chemotype, represented by compound **1**, for which anti-MbtI properties had never been previously reported. These preliminary observations could provide the basis for the design of new inhibitors.

Molecular modeling studies allowed to identify a reliable binding mode of **1** within the MbtI catalytic site and to rationalize the preliminary SAR data based on the predicted-ligand protein interactions, thus paving the way for lead optimization studies focused on the development of more potent analogues. It should be highlighted that **1** retains some of the interactions evidenced by **I** in the crystallographic conformation inside the binding pocket, but it also allows to evidence a new possible role for the oxygen backbone of G270, thus identifying a new anchoring point for the design of MbtI inhibitors. Indeed, all compounds endowed with a considerable activity showed an interaction with G270 through their 7-hydroxyl group in their predicted binding mode. Finally, the importance of the carboxylic group for the inhibitory activity of the ligands was demonstrated by the inactivity of the methyl ester **1b**, for which a completely different binding mode, lacking the key ligand–protein interactions, was predicted.

Overall, these new findings could enhance the possible perspectives in antitubercular drug discovery.

## Figures and Tables

**Table 1:** In vitro activity of compounds **1**–**5** and **I**.

Code	Structure	MbtI Residual Activity at 100 μM	MbtI IC_50_ (μM)
**I**	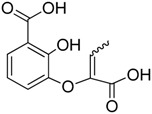	11.0 ± 3.9	11.6 ± 2.4
**1**	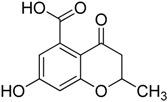	23.3 ± 3.4	55.8 ± 4.2
**1b**	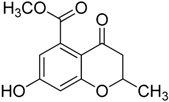	>75	ND
**2**	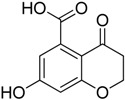	28.1 ± 2.9	61.3 ± 5.3
**3**	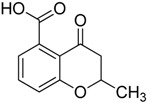	>75	ND
**4**	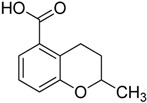	>75	ND
**5**	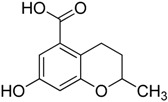	50.1 ± 7.1	ND

ND: not determined.

## Supplementary Materials

The supplementary materials are available online. The IR, MS and NMR spectra of compounds are provided. Figure S1: minimized average structure of compound **1**; Figure S2: predicted binding mode of compound **1b** into MbtI catalytic site.
