# Supplementary material for: New Chromane-Based Derivatives as Inhibitors of Mycobacterium tuberculosis Salicylate Synthase (MbtI): Preliminary Biological Evaluation and Molecular Modeling Studies
Source: Molecules. 2018 Jun 21;23(7):1506. doi: 10.3390/molecules23071506 (PMC6099841; doi:10.3390/molecules23071506)
Supplement: Supplementary file 1 [file molecules-23-01506-s001.zip › molecules-313198-supplementary.docx]

New Chromane-Based Derivatives as Inhibitors of *Mycobacterium tuberculosis* Salicylate Synthase (MbtI): Preliminary Biological Evaluation and Molecular Modeling Studies

Elena Pini ^1,†^, Giulio Poli ^2,†^, Tiziano Tuccinardi ^2^, Laurent Roberto Chiarelli ^3^, Matteo Mori ^1^,
Arianna Gelain ^1^, Luca Costantino ^4^, Stefania Villa ^1^, Fiorella Meneghetti ^1,^* and
Daniela Barlocco ^1^

^1^ Dipartimento di Scienze Farmaceutiche, Università degli Studi di Milano, Via L. Mangiagalli 25, 20133 Milano, Italy; [elena.pini@unimi.it](mailto:elena.pini@unimi.it) (E.P.); [matteo.mori@unimi.it](mailto:matteo.mori@unimi.it) (M.M.); [arianna.gelain@unimi.it](mailto:arianna.gelain@unimi.it) (A.G.); [stefania.villa@unimi.it](mailto:stefania.villa@unimi.it) (S.V.); [daniela.barlocco@unimi.it](mailto:daniela.barlocco@unimi.it) (D.B.)

^2^ Dipartimento di Farmacia, Università di Pisa, via Bonanno 6, 56126 Pisa, Italy; [giulio.poli@unipi.it](mailto:giulio.poli@unipi.it) (G.P.); [tiziano.tuccinardi@unipi.it](mailto:tiziano.tuccinardi@unipi.it) (T.T.)

^3^ Dipartimento di Biologia e Biotecnologie "Lazzaro Spallanzani", Università degli Studi di Pavia, via Ferrata 9, 27100 Pavia, Italy; laurent.chiarelli@unipv.it

^4^ Dipartimento Scienze della Vita, Università degli Studi di Modena e Reggio Emilia, via Campi 103, 41121 Modena, Italy; luca.costantino@unimore.it

***** Correspondence: fiorella.meneghetti@unimi.it; Tel.: +39-0250319306

† These authors contributed equally.

**Supporting Information**

| **1.** | **Analytical data for compound 1** | |  |
| --- | --- | --- | --- |
|  | **1.1** | FTIR spectrum……………………………………………………...... | S4 |
|  | **1.2** | ^1^H NMR spectrum…………………………………………………… | S4 |
|  | **1.3** | ^13^C NMR spectrum………………………………………………...… | S5 |
|  | **1.4** | HSQC spectrum……………………………………………………… | S5 |
|  | **1.5** | ESI-MS spectrum……………………………………………………. | S6 |
|  | **1.6** | HRMS spectrum……………………………………………………... | S6 |
| **2.** | **Analytical data for compound 1a** | |  |
|  | **2.1** | FTIR spectrum……………………………………………………...... | S8 |
|  | **2.2** | ^1^H NMR spectrum…………………………………………………… | S8 |
|  | **2.3** | ESI-MS spectrum…………………………………………………..... | S9 |
| **3.** | **Analytical data for compound 1b** | |  |
|  | **3.1** | FTIR spectrum……………………………………………………...... | S10 |
|  | **3.2** | ^1^H NMR spectrum…………………………………………………… | S10 |
|  | **3.3** | ^13^C NMR spectrum…………………………………………………... | S11 |
|  | **3.4** | ESI-MS spectrum……………………………………………………. | S11 |
|  | **3.5** | HRMS spectrum……………………………………………………... | S12 |
| **4.** | **Analytical data for compound 2** | |  |
|  | **4.1** | FTIR spectrum……………………………………………………...... | S13 |
|  | **4.2** | ^1^H NMR spectrum…………………………………………………… | S13 |
|  | **4.3** | ^13^C NMR spectrum…………………………………………………... | S14 |
|  | **4.4** | ESI-MS spectrum……………………………………………………. | S14 |
|  | **4.5** | HRMS spectrum……………………………………………………... | S15 |
| **5.** | **Analytical data for compound 3** | |  |
|  | **5.1** | FTIR spectrum……………………………………………………...... | S16 |
|  | **5.2** | ^1^H NMR spectrum…………………………………………………… | S16 |
|  | **5.3** | ^13^C NMR spectrum…………………………………………………... | S17 |
|  | **5.4** | ESI-MS spectrum…………………………………………………..... | S17 |
|  | **5.5** | HRMS spectrum……………………………………………………... | S18 |
| **6.** | **Analytical data for compound 4** | |  |
|  | **6.1** | FTIR spectrum……………………………………………………...... | S19 |
|  | **6.2** | ^1^H NMR spectrum…………………………………………………… | S19 |
|  | **6.3** | ^13^C NMR spectrum…………………………………………………... | S20 |
|  | **6.4** | COSY spectrum…………………………………………………….... | S20 |
|  | **6.5** | ESI-MS spectrum …………………………………………………….. | S21 |
|  | **6.6** | HRMS spectrum……………………………………………………... | S21 |
| **7.** | **Analytical data for compound 5** | |  |
|  | **7.1** | FTIR spectrum……………………………………………………...... | S23 |
|  | **7.2** | ^1^H NMR spectrum…………………………………………………… | S23 |
|  | **7.3** | ^13^C NMR spectrum…………………………………………………... | S24 |
|  | **7.4** | ESI-MS spectrum…………………………………………………….. | S24 |
|  | **7.5** | HRMS spectrum……………………………………………………... | S25 |
| **8.** | **Compared binding modes of compounds 1 and I** | | S26 |
| **9.** | **Predicted binding mode of compound 1b** | | S27 |

1. **Analytical data for compound 1**
   1. **FTIR spectrum**

**1.2 ^1^H NMR spectrum**

**1.3 ^13^C NMR spectrum**

**1.4 HSQC spectrum**

**1.5 ESI-MS spectrum**


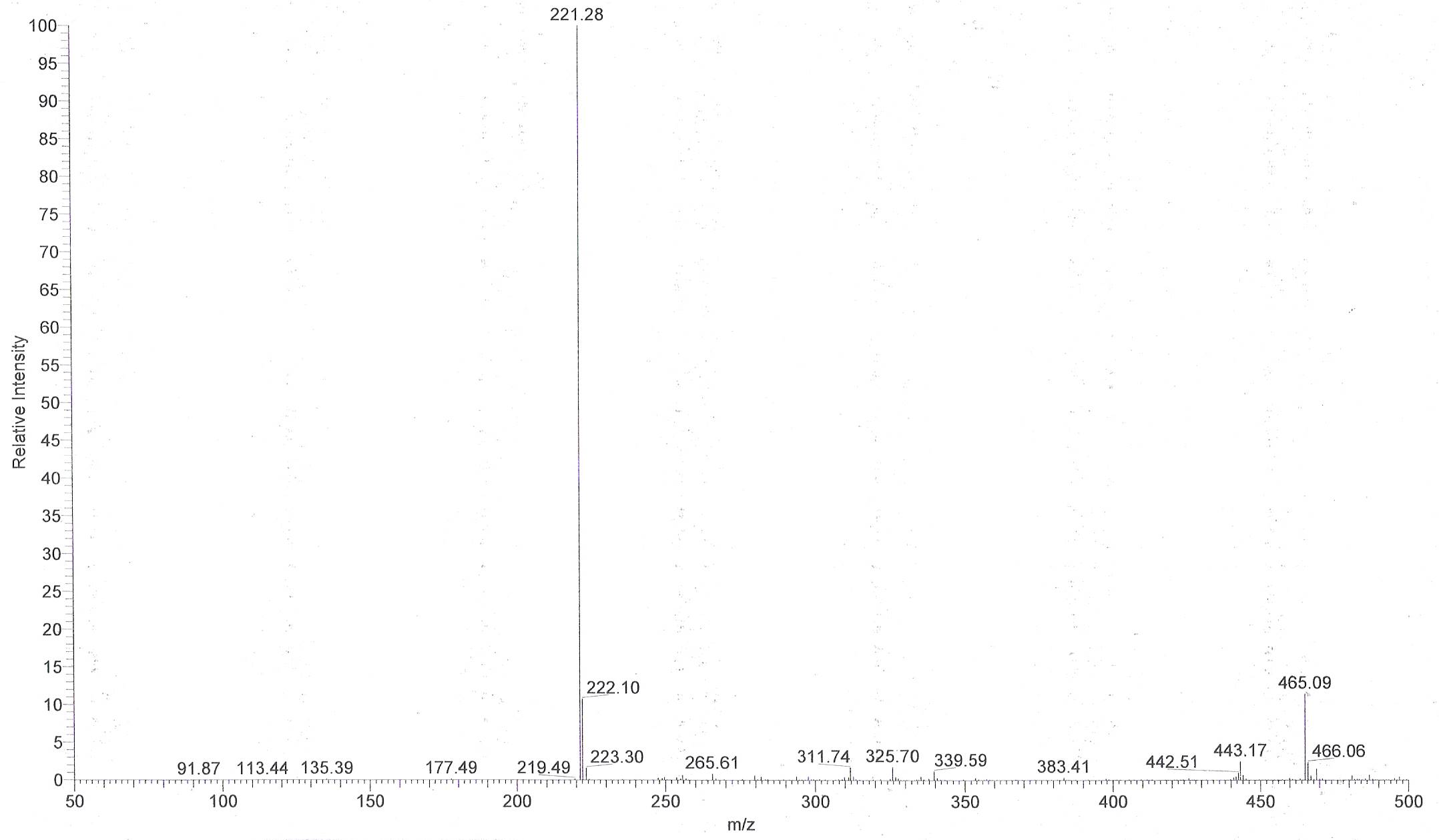


**1.6 HRMS spectrum**

**
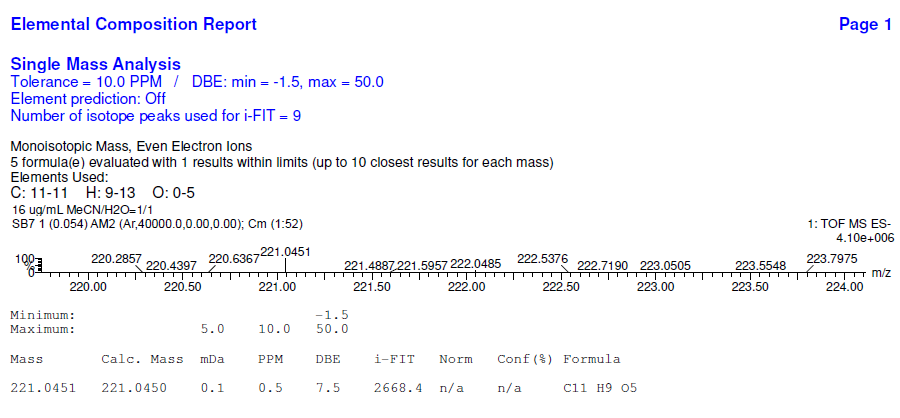
**

**
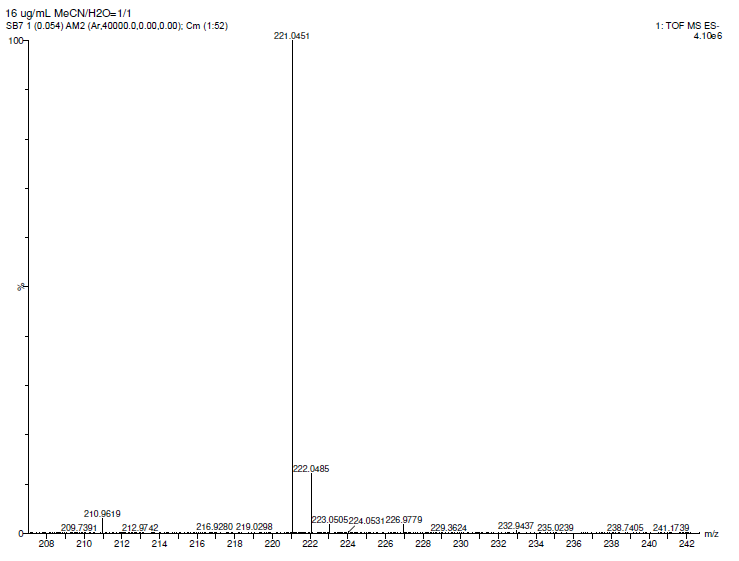
**

1. **Analytical data for compound 1a**
   1. **FTIR spectrum**

**2.2 ^1^H NMR spectrum**

**2.3 ESI-MS spectrum**


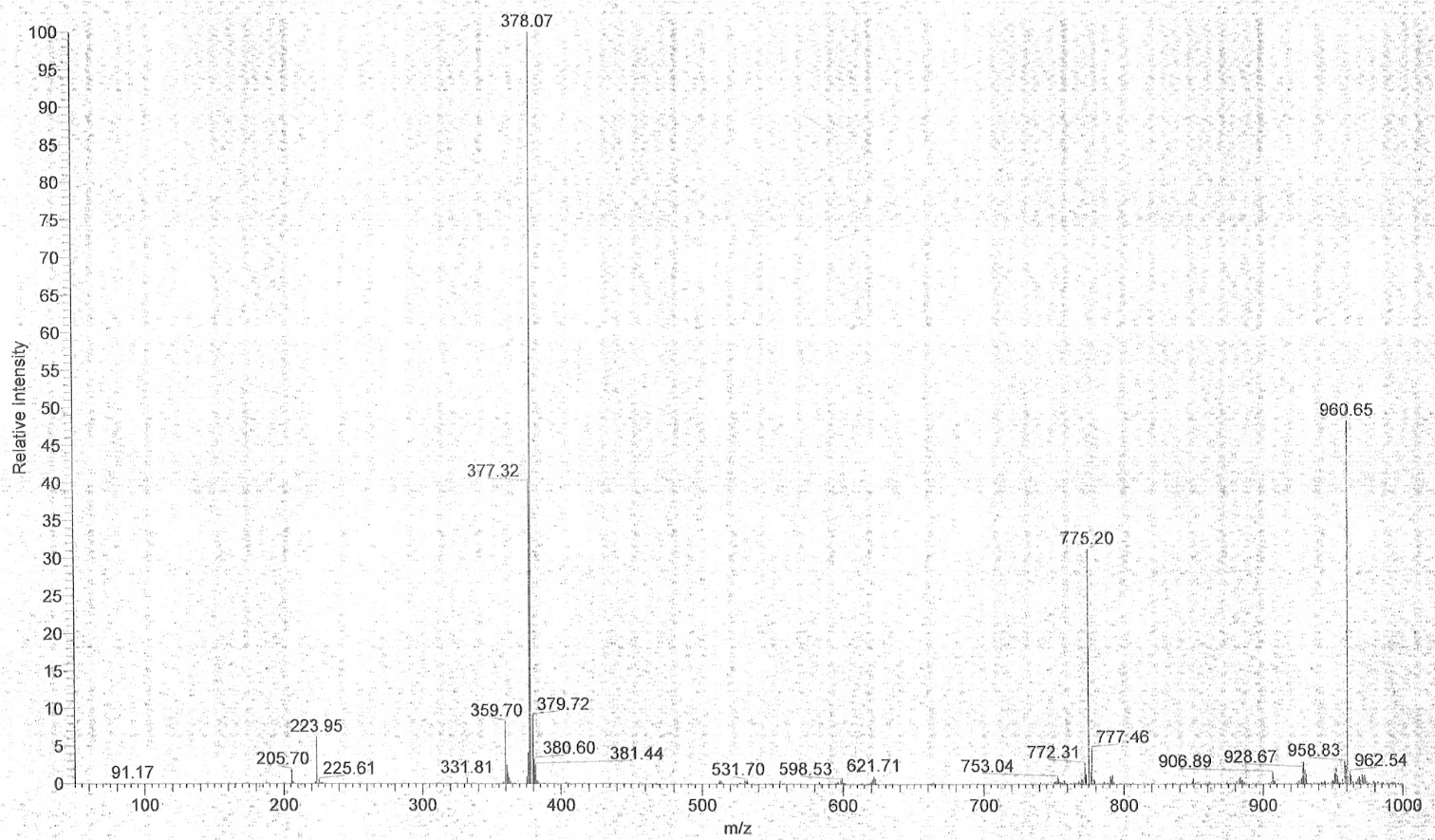


1. **Analytical data for compound 1b**
   1. **FTIR spectrum**

**3.2 ^1^H NMR spectrum**

**3.3 ^13^C NMR spectrum**

**3.4 ESI-MS spectrum**

**
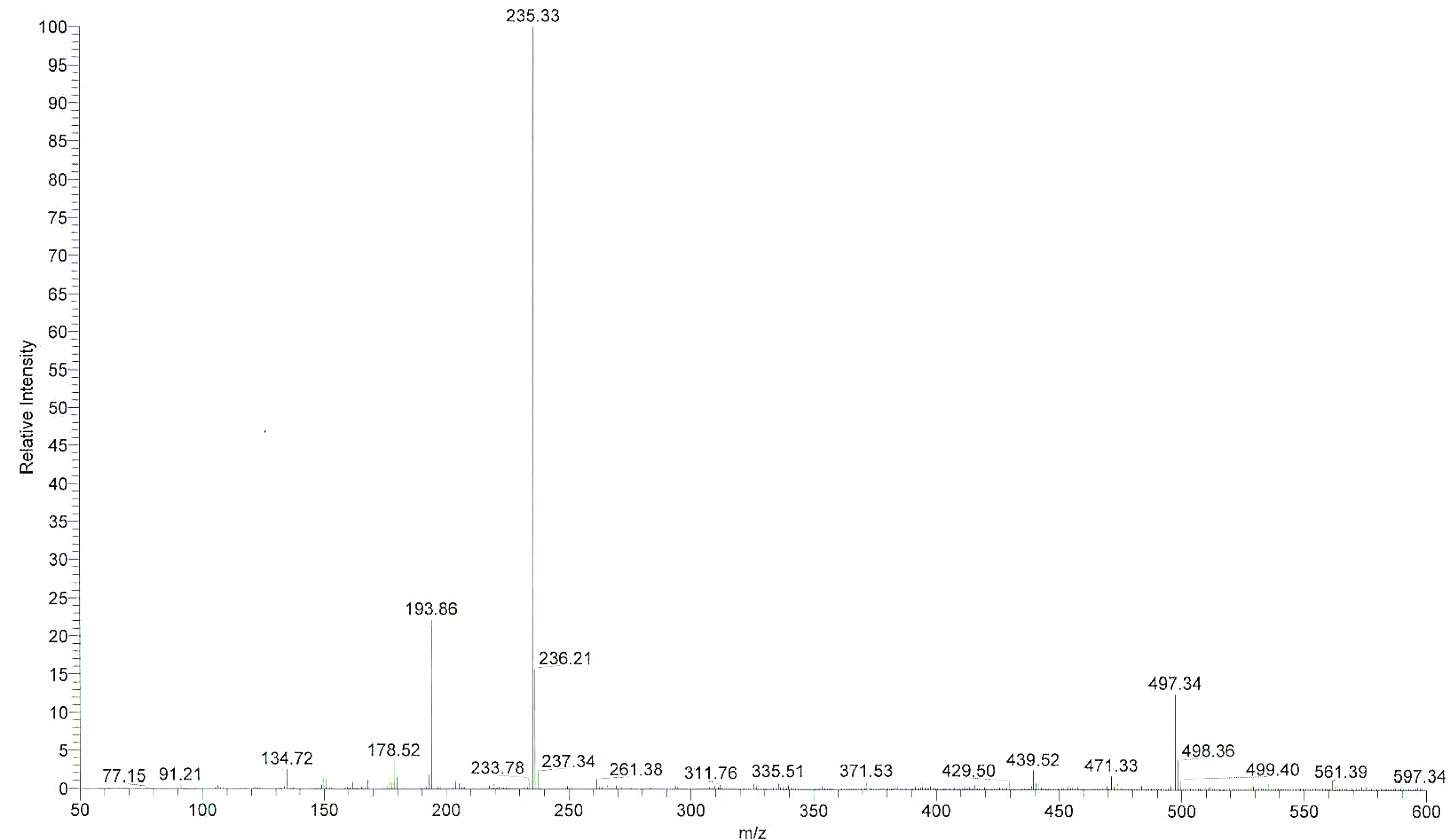
**

**3.5 HRMS spectrum**

**
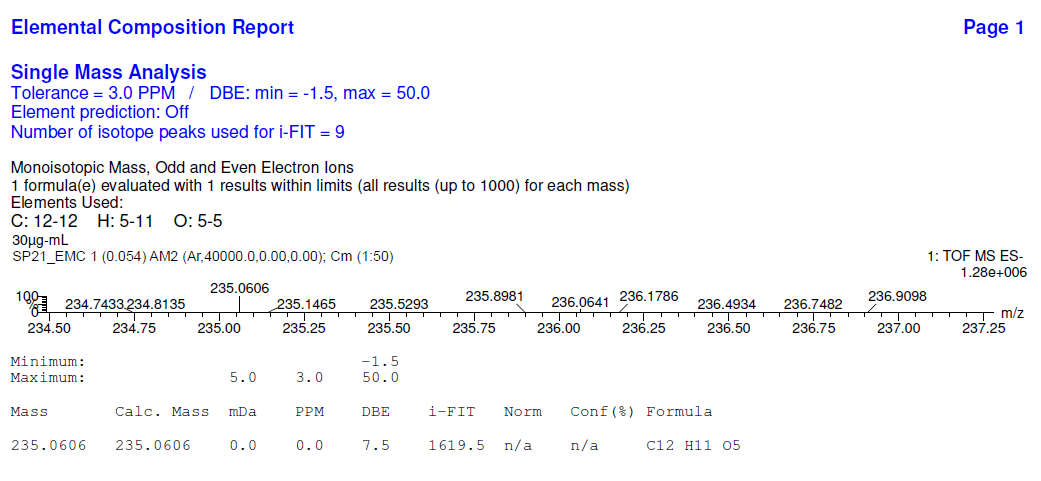
**

**
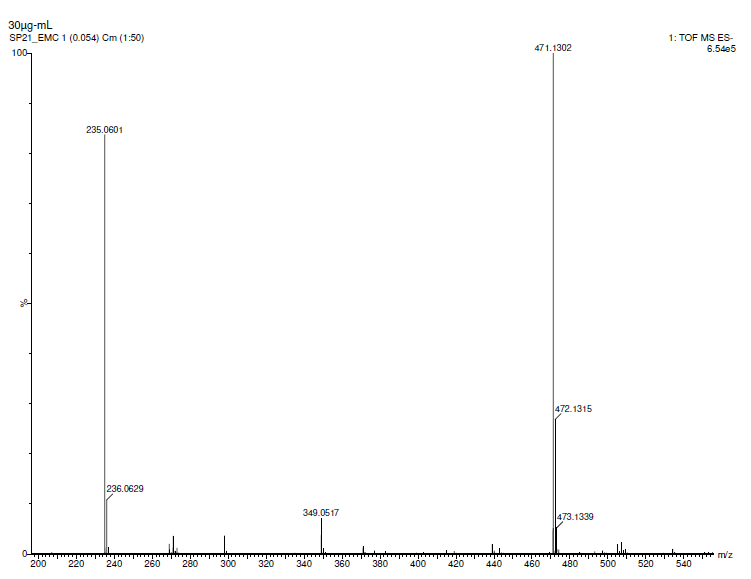
**

1. **Analytical data for compound 2**
   1. **FTIR spectrum**

**4.2 ^1^H NMR spectrum**

**4.3 ^13^C NMR spectrum**

**4.4 ESI-MS spectrum**


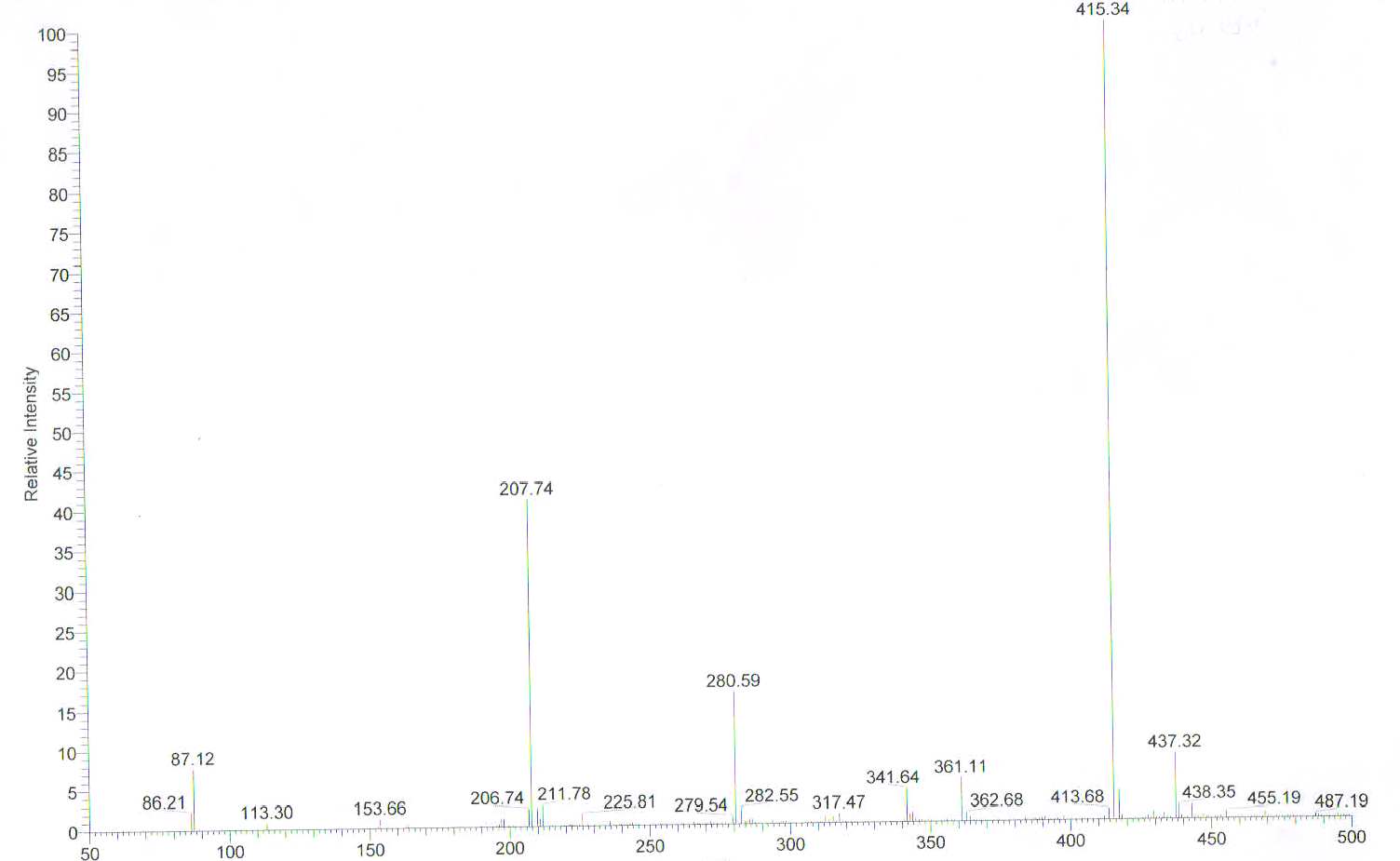


**4.5 HRMS spectrum**

**
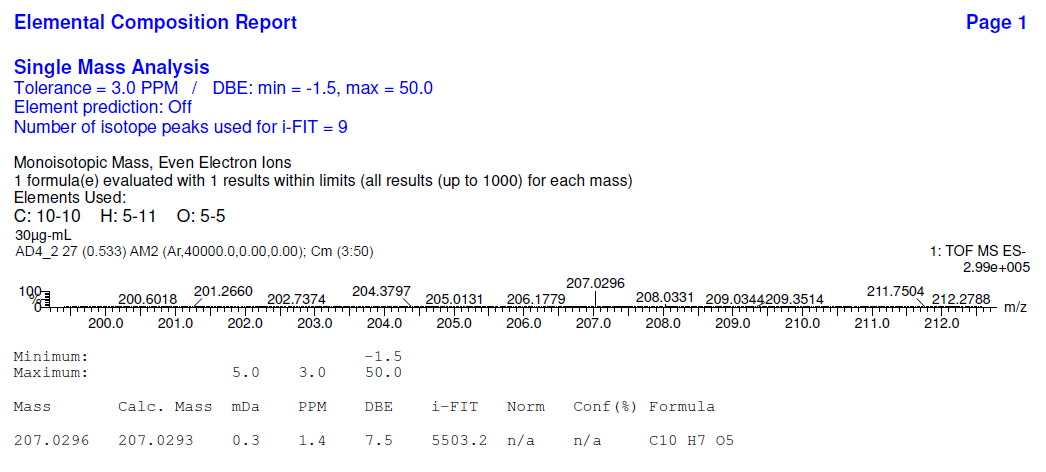
**

**
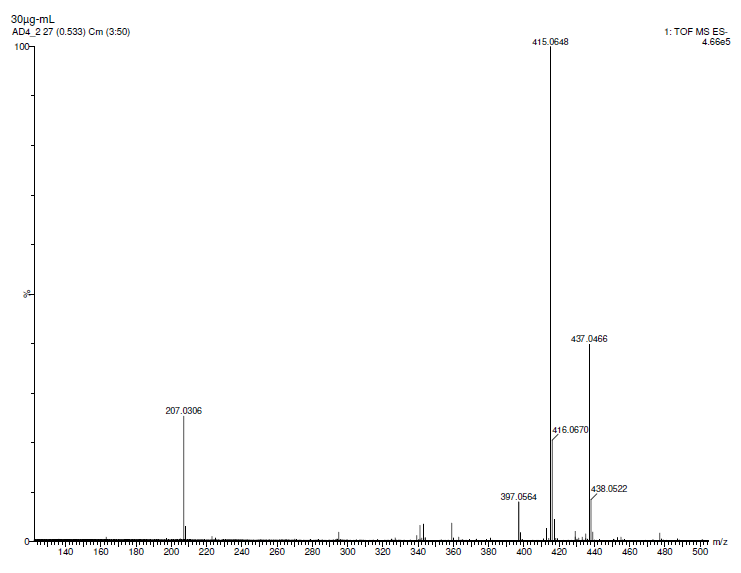
**

1. **Analytical data for compound 3**
   1. **FTIR spectrum**

**5.2 ^1^H NMR spectrum**

**5.3 ^13^C NMR spectrum**

**5.4 ESI-MS spectrum**

**
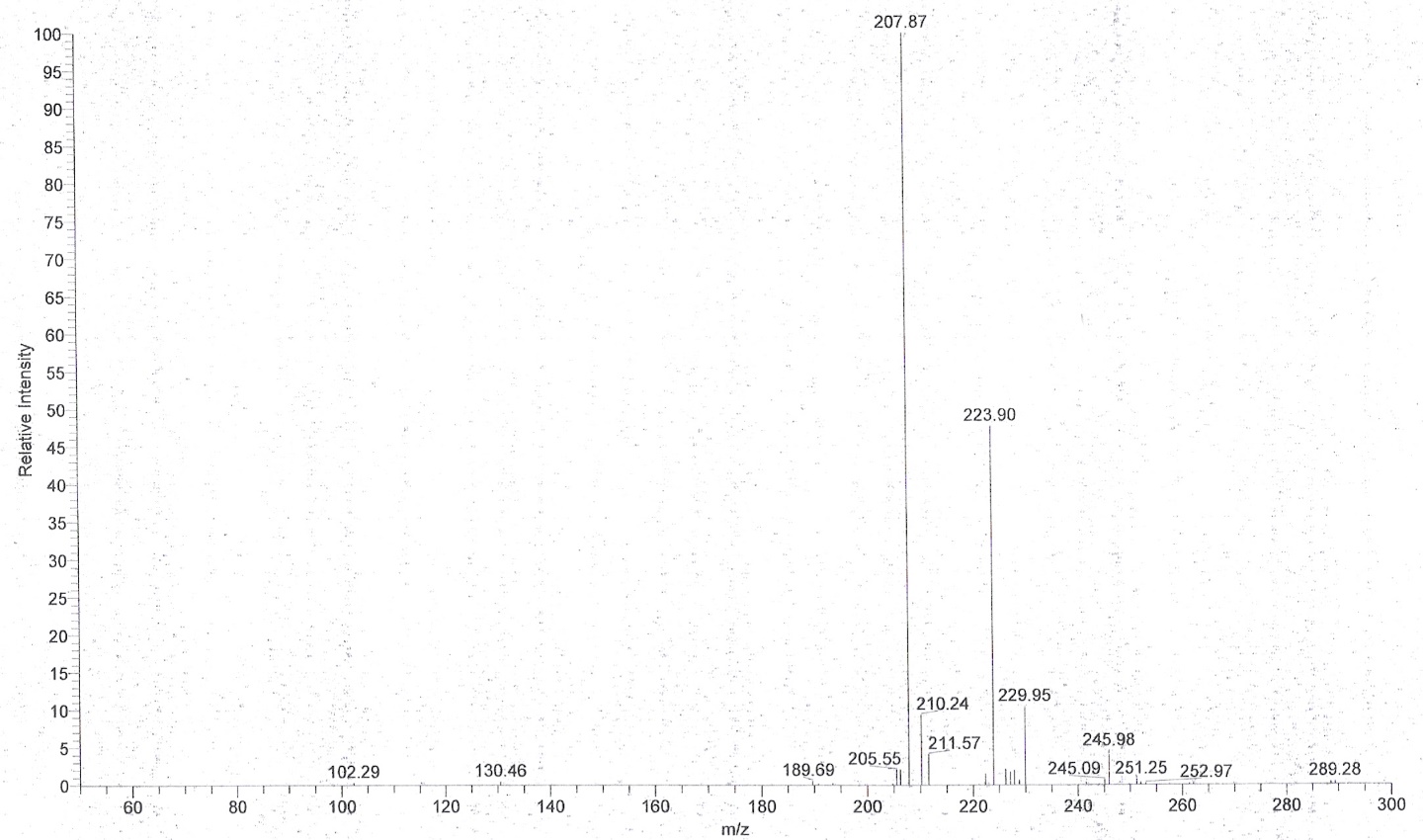
**

**5.5 HRMS spectrum**

**
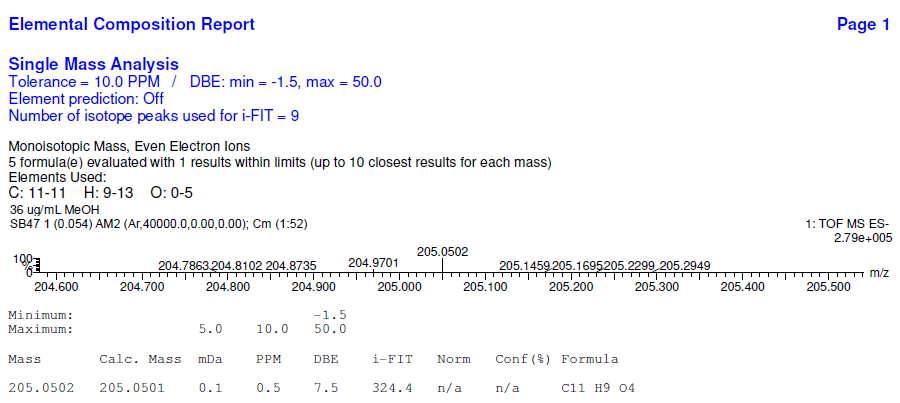
**

**
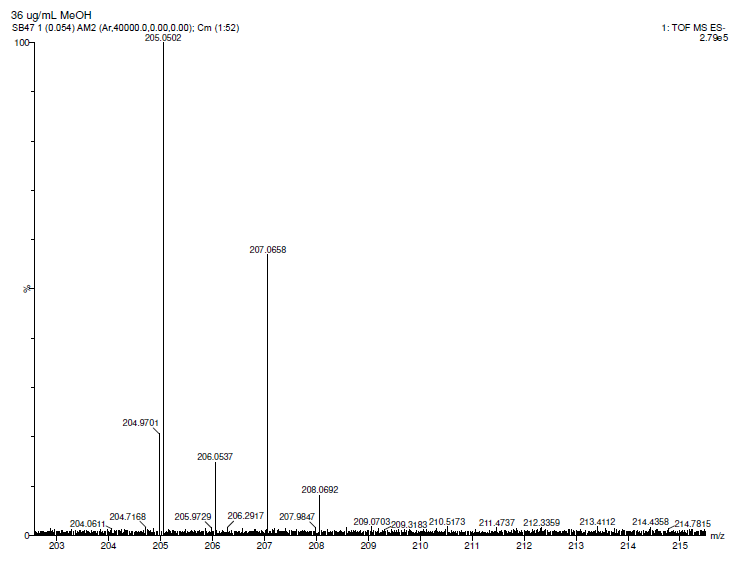
**

1. **Analytical data for compound 4**
   1. **FTIR spectrum**

**6.2 ^1^H NMR spectrum**

**6.3 ^13^C NMR spectrum**

**6.4 COSY spectrum**

**6.5 ESI-MS spectrum**

**
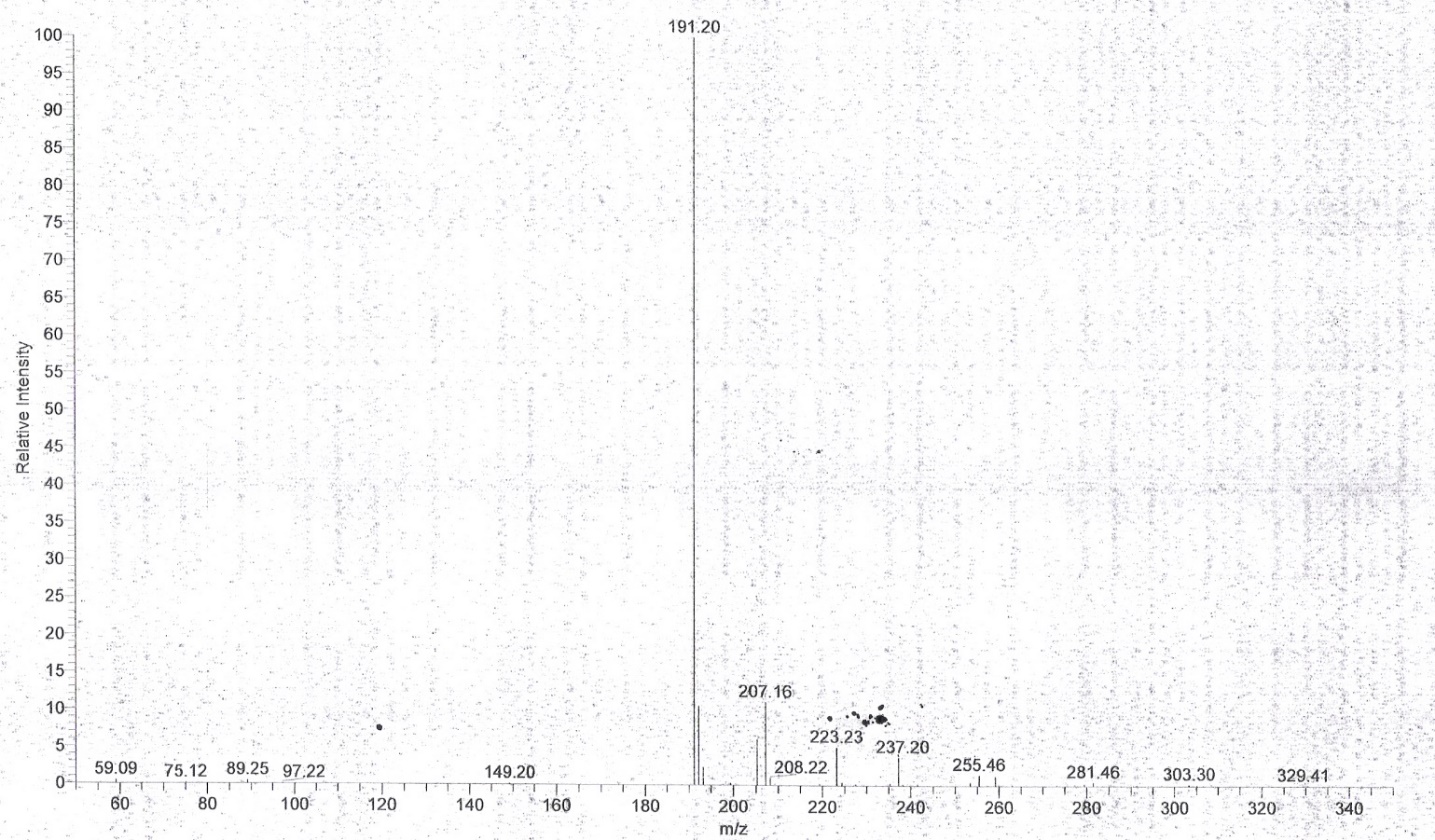
**

**6.6 HRMS spectrum**

**
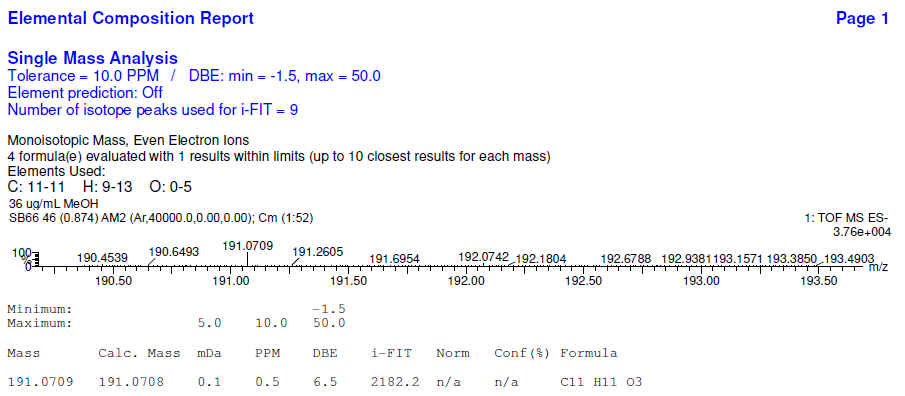
**

**
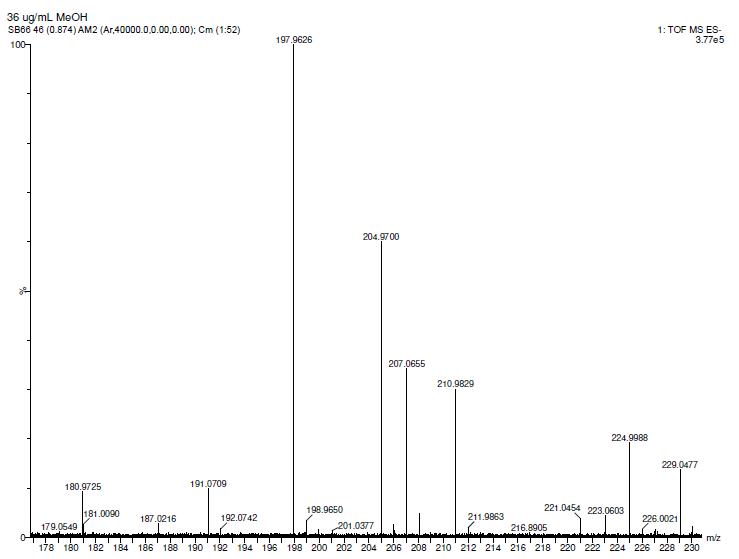
**

1. **Analytical data for compound 5**
   1. **FTIR spectrum**

**
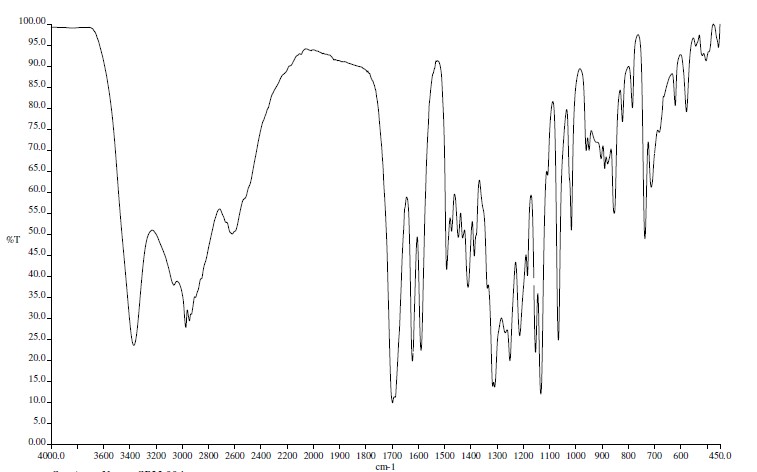
**

**7.2 ^1^H NMR spectrum**

**7.3 ^13^C NMR spectrum**

**7.4 ESI-MS spectrum**

**
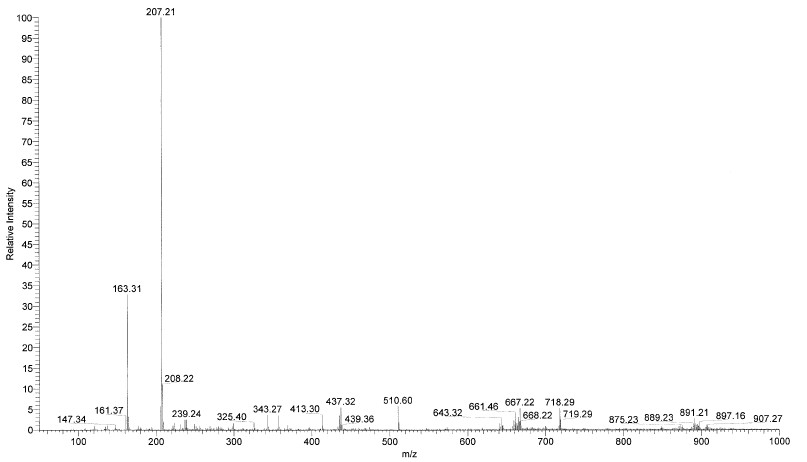
**

**7.5 HMRS spectrum**

**
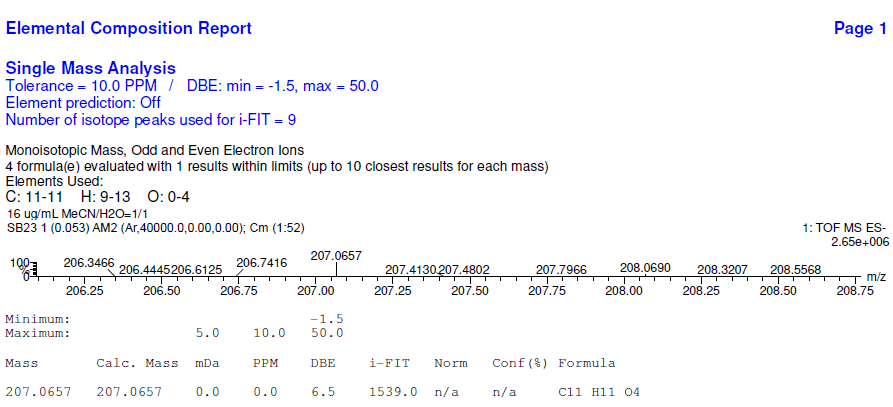
**

**
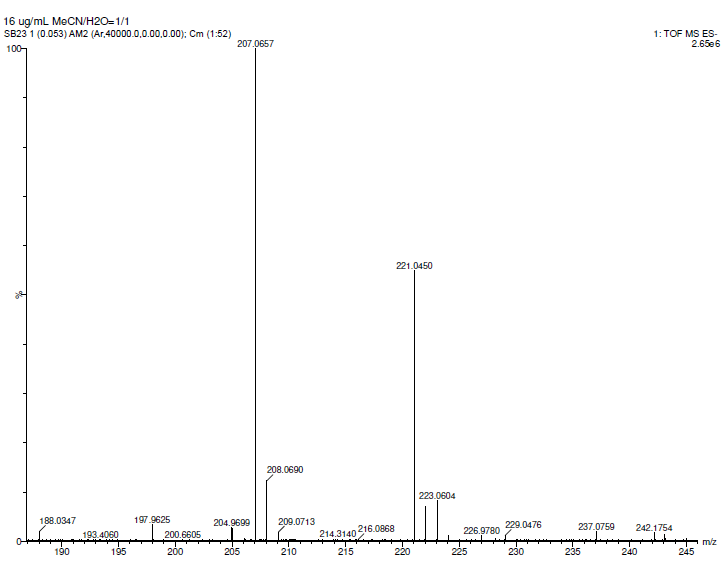
**

1. **Compared binding modes of compounds 1 and I**

**
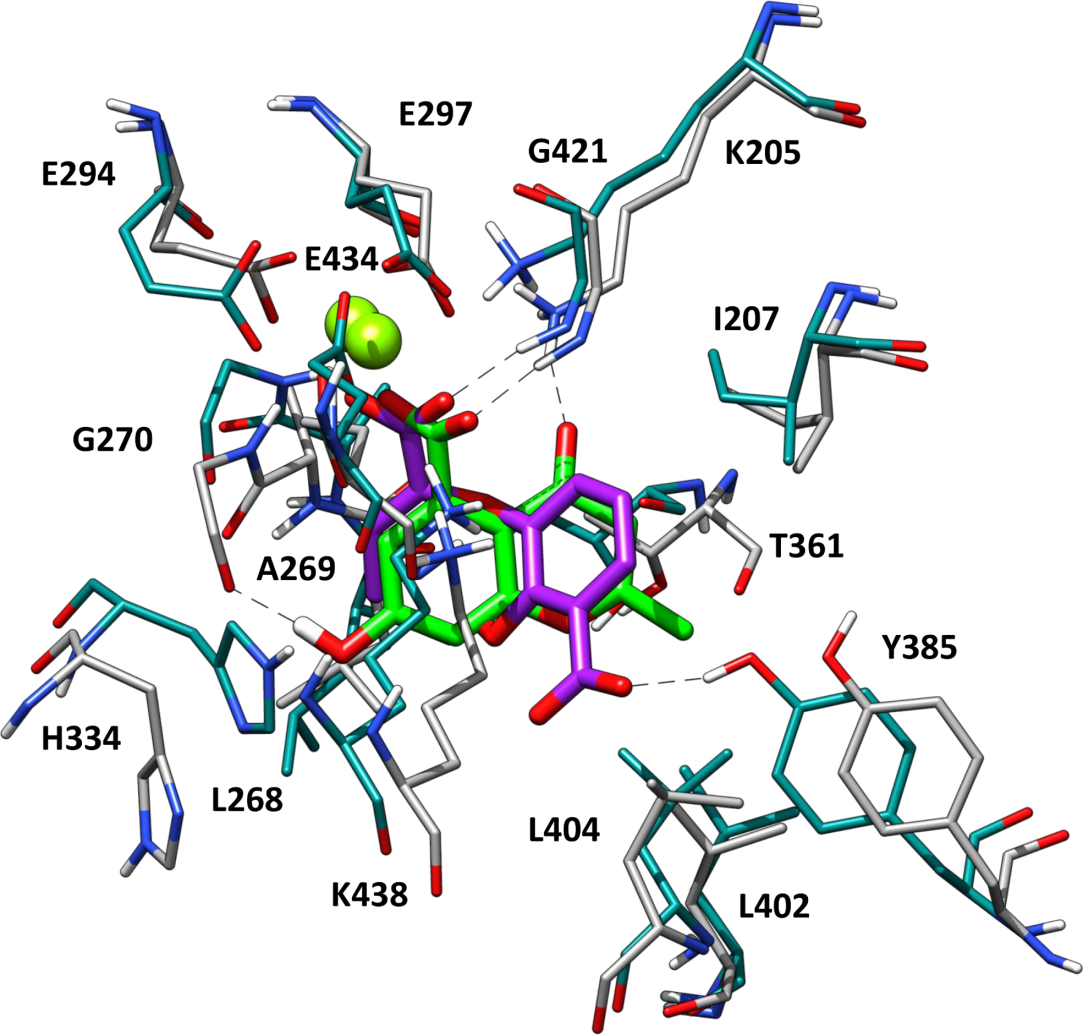
**

**Figure S1.** Minimized average structure of compound **1** (green) docked into MbtI catalytic site (gray), superimposed with the reference MbtI-compound **I** complex (dark cyan and purple, respectively), subjected to the same molecular dynamics simulation protocol.

1. **Predicted binding mode of compound 1b**


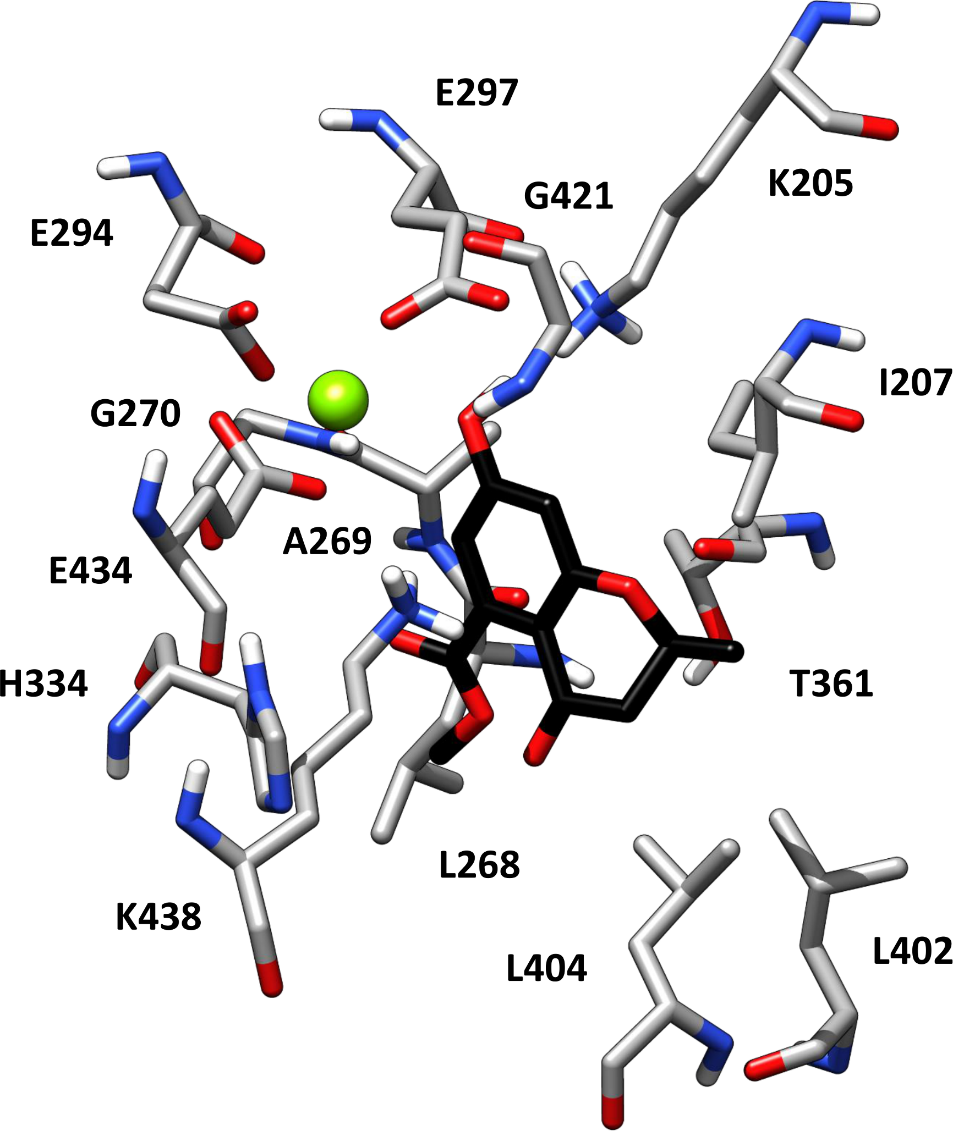


**Figure S2.** Predicted binding mode of compound **1b** into MbtI catalytic site.
